# Regional Changes in Brain Biomolecular Markers in a Collagen-Induced Arthritis Rat Model

**DOI:** 10.3390/biology13070516

**Published:** 2024-07-10

**Authors:** Aletta M. E. Millen, Tshiamo T. Maluleke, Leandrie Pienaar, Farhanah N. Sallie, Radhini Veerappan, Per E. Andrén, Sooraj Baijnath

**Affiliations:** 1 Wits Integrated Molecular Physiology Research Initiative, Wits Health Consortium (PTY) Ltd., University of the Witwatersrand, Johannesburg 2191, South Africa; tiiso.maluleke@wits.ac.za (T.T.M.); 1836318@students.wits.ac.za (L.P.); 1894684@students.wits.ac.za (F.N.S.); radhini.veerappan@wits.ac.za (R.V.); sooraj.baijnath@wits.ac.za (S.B.); 2 School of Physiology, Faculty of Health Sciences, University of The Witwatersrand, Johannesburg 2191, South Africa; 3 Department of Pharmaceutical Biosciences, Spatial Mass Spectrometry, Science for Life Laboratory, Uppsala University, 75121 Uppsala, Sweden; per.andren@uu.se

**Keywords:** collagen-induced arthritis (CIA), inflammation, brain, neurotrophic factors, neurotransmitters, neuroinflammation

## Abstract

**Simple Summary:**

Auto-immune disorders, such as rheumatoid arthritis, are characterized by high levels of pro-inflammatory cytokines. Continued exposure to high levels of systemic inflammation has several other health consequences, including negative effects on organs such as the brain. These detrimental effects may result in mood and neurological disorders. However, the exact mechanisms through which inflammation can result in mood disturbances and neurological disorders are not well known and are difficult to study in humans. The collagen-induced arthritis (CIA) rat model has been used to mimic human autoimmune disorders and systemic inflammation. In this study, we used the CIA model to gain a better understanding of the role of inflammation on the processes that may be involved in mood and neurological disorders, by looking at several molecular markers in different brain regions. Our results showed that all the brain regions studied in the CIA rats had high levels of local inflammation, which may have resulted in cell death in these areas. We also showed that the markers associated with neuron health and neurotransmitters implicated in mood disturbances were dysregulated in some, but not all brain regions. These results suggest that inflammation induced by autoimmune disorders may result in neuroinflammation that could result in neurodegeneration.

**Abstract:**

Background: The effects of collagen-induced arthritis (CIA), a model of systemic inflammation, on brain regional molecular markers associated with neurological disorders are uncertain. Objective: This study investigated the brain regional molecular changes in markers associated with inflammation and neuronal dysfunction in a CIA model. Methods: Fourteen male Sprague Dawley rats were divided into control (n = 5) or CIA (n = 9) groups. 10 weeks after CIA induction, brain tissue was collected. Brain regional mRNA expression of inflammatory markers (*IL-1β* and *IL-6*), apoptotic markers (*BAX* and *Bcl2*) and neurotrophic factors (*BDNF*, *CREB* and *TrkB*) was determined. Monoamine distribution and abundance in different brain regions were determine by mass spectrometry imaging (MSI). Results: Neuroinflammation was confirmed in the CIA group by increased *IL-β* mRNA expression, concurrent with an increased *BAX/Bcl2* ratio. The mRNA expression of *CREB* was increased in the midbrain and hippocampus while *BDNF* was increased and *TrkB* was decreased across all brain regions in CIA compared to control animals. Serotonin was decreased in the midbrain and hippocampus while dopamine was decreased in the striatum of CIA rats, compared to controls. Conclusion: CIA resulted in neuroinflammation concurrent with an apoptotic state and aberrant expression of neurotrophic factors and monoamines in the brain, suggestive of neurodegeneration.

## 1. Introduction

The collagen-induced arthritis (CIA) rodent model has been extensively used in the study of autoimmune disorders [1] and has been considered the gold standard for rheumatoid arthritis (RA) studies [2]. RA is particularly known for its extra-articular manifestations, which are believed to be driven by the underlying systemic inflammation [3]. Hence, the use of CIA rodent models has been important in improving our understanding of systemic-inflammation-induced pathophysiological changes that could be translated into several clinical characteristics. Indeed, we and others have used the CIA model to investigate the mechanisms of inflammation-induced cardiovascular disease [4,5,6,7,8,9,10], pulmonary disorders [11], metabolic disturbances [12] and several other disorders. Of particular importance for this study is the previously reported neurological dysfunction in the CIA model [13,14,15]. Indeed, systemic inflammation has been identified as one of the major drivers in the pathogenesis of several neurological disorders, including mood disorders [16].

It is well accepted that patients with systemic inflammatory disorders, such as RA, are at increased risk for developing mood disturbances [17,18]. Several clinical symptoms consistent with mood disorders have been reported in patients with RA [19]. Moreover, a strong association between circulating inflammatory cytokines and depressive-like symptoms have been shown in the general population [20] and in patients with auto-immune disorders [21]. The development of mood disorders is multifactorial, with several pathophysiological mechanisms proposed. These mechanisms include monoamine dysregulation, deficiency of neurotrophic factors, excitatory/inhibitory imbalance, hypothalamic–pituitary–adrenal (HPA) axis dysregulation and neuroinflammation [22]. This wide range of pathological processes makes mood disorders increasingly difficult to manage in a public healthcare setting, as seen in the increasing prevalence of treatment-resistant depression, a rapidly rising public health concern [23]. Interestingly, neuroinflammation has been suggested as a point of convergence of these multiple theories and may be a possible treatment strategy in managing mood disorders in inflammatory conditions [19]. However, the molecular mechanisms through which inflammation contributes to the development of neuronal dysfunction are currently under investigation.

Although other rodent models of systemic inflammation using adjuvant-induced arthritis (AIA) have shown neurological changes [24,25], limited studies are available using the CIA model. Nevertheless, previous studies have shown that CIA resulted in neurobehavioral changes including anxiety-like symptoms as measured by the elevated-plus and elevated-zero maze tests and cognitive impairment as measured by the Morris water maze, the olfactory social memory test and the passive avoidance test [13,26]. The depressive-like symptoms measured by these behavioural tests have been related to common phenotypes observed in animal models of neuropathophysiology.

It is well established that immune system dysregulation impacts the brain, possibly via several distinct pathways [27]. In this regard, circulating cytokines have been associated with HPA axis dysregulation, which is strongly related to mood disorders [28]. In addition, in the CIA model, increased inflammatory cytokines have shown to increase the permeability of the blood–brain barrier [29], which may result in inflammatory cells migrating from the periphery into the brain. These pro-inflammatory cytokines have been implicated in the activation of indoleamine 2,3-dioxygenase (IDO) [30], an enzyme that catalyses the rate-limiting first step in tryptophan catabolism and the kynurenine pathway, leading to decreased synaptic availability of serotonin. This is important, since monoamine dysregulation is one of the most well-accepted mechanisms underlying neurological disorders [22]. However, whether neurotransmitter signalling is disrupted in the CIA model is currently unknown. Furthermore, activation of inflammatory signalling pathways in the brain has been associated with decreased brain-derived neurotrophic factor (BDNF) and cyclic AMP response binding element (CREB) expression [31,32]. These biomarkers are transcription factors associated with neuronal survival and plasticity [31]. Dysregulated BDNF and CREB expression have consistently been reported in neurodegenerative diseases, such as Parkinson’s disease, multiple sclerosis and Huntington’s disease [33], and in mood disorders associated with anxiety and depressive-like symptoms [31,34].

In rodents, treatment with anti-rheumatic drugs has shown improvements in CIA-induced anxiety-like symptoms and cognitive impairments, further suggesting a strong association between inflammation and the development of neurological effects [35]. However, the brain is a highly compartmentalized organ with various regions controlling different biomolecular processes, which are affected to varying degrees in brain disorders. Whether biomolecular markers of neurological disorders are differentially altered in distinct brain regions is currently uncertain in the CIA model. Therefore, in this study we aimed to investigate the brain-region-specific changes in inflammatory markers, neurotropic and apoptotic factors and neurotransmitter abundance in a CIA rodent model.

## 2. Methods

### 2.1. Study Design

Male Sprague Dawley rats (n = 14) were obtained from the Wits Animal Research Facility at the University of Witwatersrand. Experimental animals were housed in polycarbonate cages (TechniPlast, Varese, Italy) under standard laboratory conditions (25 °C ± 1 °C, 24–26% humidity) with a 12 h light/dark cycle. Animals were allowed *ad libitum* access to standard rat chow and normal drinking water throughout the study and allowed to acclimatize for 7–14 days prior to any experimental procedures. Rats in the control group (n = 5) received daily saline injections (0.1 mL, i.p). To induce systemic inflammation, rats in the CIA group (n = 9) were inoculated with 0.2 mL (200 μg) of the bovine type II collagen emulsified in incomplete Fruend’s adjuvant by subcutaneous injection at the base of the tail, followed by a 0.1 mL (100 μg) booster injection 7 days after the first immunization, as previously described [5]. To ensure a high incidence of inflammation, 21 days after the initial immunization, rats in the CIA group received intraperitoneal injections of lipopolysaccharide at a dosage of 50 µg/kg. The CIA model is a well-accepted rodent model of autoimmunity where animals develop severe autoimmune-induced polyarticular arthritis in response to the administration of type II collagen [1]. Seven weeks later, the rats were terminated, and their brains were surgically removed. Brain tissue was dissected into two hemispheres: The left hemisphere was used for mass spectrometry imaging (MSI). The right hemisphere was further dissected into different brain regions for molecular analysis. Brain tissue samples were frozen in liquid nitrogen and stored at −80 °C until further analysis.

### 2.2. Biomolecular Marker Gene Expression

For gene expression analysis, individual brain regions were homogenized in lysis buffer using probe sonication (Fisher Scientific, Waltham, MA, USA). Total RNA was extracted from tissue homogenates using the Illustra Minispin RNA extraction kit (GE Healthcare, Chicago, IL, USA). cDNA was then synthesized using the SuperScript VILO cDNA synthesis kit (ThermoFisher, Waltham, MA, USA). The quality and concentration of RNA and cDNA were determined using a NanoDrop One^C^ (Thermo Scientific, Waltham, MA, USA) and were stored at −80 °C until required.

Commercially available TaqMan assays were used to determine *IL-1β*, *IL-6*, *BAX*, *Bcl2*, *TrKB*, *BDNF* and *CREB* mRNA expressions using RT-PCR (ThermoScientific, Waltham, MA, USA), with *Tbp* as the housekeeping gene. Using RefFinder analysis, *Tbp* was considered the most stable and reliable reference gene. Amplification of the genes of interest was performed on a StepOne Real-Time PCR System (Applied Biosystems, Foster City, CA, USA). Fold changes in mRNA expression were determined using the 2^−ΔΔCT^ method [36].

### 2.3. Mass Spectrometry Imaging Neurotransmitter Analysis

Prior to MALDI-MSI analysis, fresh-frozen brain hemispheres were sectioned using a Leica 1900 UV cryomicrotome (Leica Microsystems, Wetzlar, Germany) and sectioned to a thickness of 12 µm (bregma—ventral striatum (+1.18 mm); hippocampus (−2.06 mm); midbrain (−3.08 mm)) before being thaw-mounted onto conductive indium titanium oxide (ITO)-coated slides (Bruker Daltonics, Bremen, Germany). Slides were stored at −80 °C prior to imaging analysis.

For MALDI-MSI of neurotransmitters, slides were removed from −80 °C storage and dried in a vacuum desiccator for 30 min at room temperature. Neurotransmitters were detected using a targeted derivatization procedure, using a fluoromethylpyridinium derivatization agent (FMP-10) that selectively targets primary amines and phenolic hydroxyl groups [37]. FMP-10 was prepared in 70% acetonitrile and 30% water (0.18 mg/mL) and uniformly applied across the tissue surface using a TM-sprayer (HTX Technologies, Chapel Hill, NC, USA), as previously described [37].

MALDI-MSI analysis was performed using a solariX 7T 2ω MALDI–Fourier-transform ion cyclotron resonance (FT-ICR) high-resolution MS system (Bruker Daltonics, Bremen, Germany), equipped with a SmartBeam II 2 kHz laser, operated in positive-ion mode and calibrated with red phosphorus prior to analysis. For MSI analysis of neurotransmitters, slides were imaged at a lateral resolution of 100 µm and spectra were collected by firing 100 laser shots per pixel, with a mass range of 150–1500 *m*/*z* and a Q1 value of 378 *m*/*z*. The time-of-flight transfer optics was 0.7 ms with a frequency of 4 MHz. Online calibration was performed using the lock mass 555.2231 *m*/*z*, an abundant ion cluster signal of the derivatization agent FMP-10.

Data were visualized using FlexImaging v5.0 software (Bruker Daltonics, Bremen, Germany) with all spectra normalized using the root mean square (RMS) method. For relative quantitation, all experiments were imported into SCiLS Lab v2019 Pro (Bruker Daltonics, Bremen, Germany) and merged into a single file. SCiLS Lab was then used to annotate regions of interest at each bregma level and to export area under the curve values for each neurotransmitter within each region.

### 2.4. Data Analysis

Data analysis was performed using SAS software (version 9.4, SAS Cary Institute, Cary, NC, USA). Data are presented as mean ± standard deviation (SD) or median (interquartile range, IQR), as appropriate. Group differences were determined using an unpaired *t*-test for normally distributed variables or Kruskal–Wallis test for non-normally distributed variables. Results are considered statistically significant if *p* < 0.05.

## 3. Results

### 3.1. CIA-Induced Neuroinflammation

**Figure 1:**
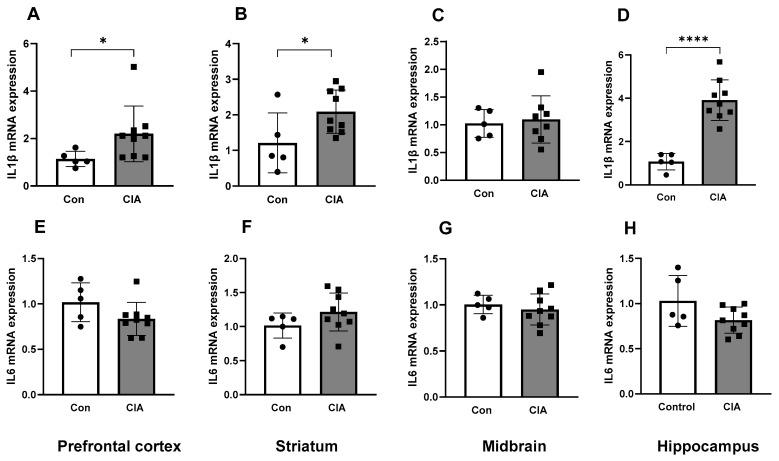
Inflammatory marker mRNA expression in different brain regions in control (n = 5) and CIA (n = 9) rats. *IL-1β* mRNA expression in the prefrontal cortex (**A**), striatum (**B**), midbrain (**C**) and hippocampus (**D**). *IL-6* mRNA expression in the prefrontal cortex (**E**), striatum (**F**), midbrain (**G**) and hippocampus (**H**). Results are presented in arbitrary units normalized for the expression of the endogenous reference gene, *Tbp*. Data are represented as mean ± SD. * *p* < 0.05, **** *p* < 0.0001.

Collagen inoculation resulted in increased *IL-1β* mRNA expression in the prefrontal cortex, striatum and hippocampus compared to controls (all *p* < 0.05, Figure 1A,B,D), but not in the midbrain (Figure 1C). Interestingly, the mRNA expression of *IL-6* did not differ between CIA rats and controls in any of the brain regions (all *p* > 0.05, Figure 1E–H).

### 3.2. Neuroinflammation-Induced Apoptosis

**Figure 2:**
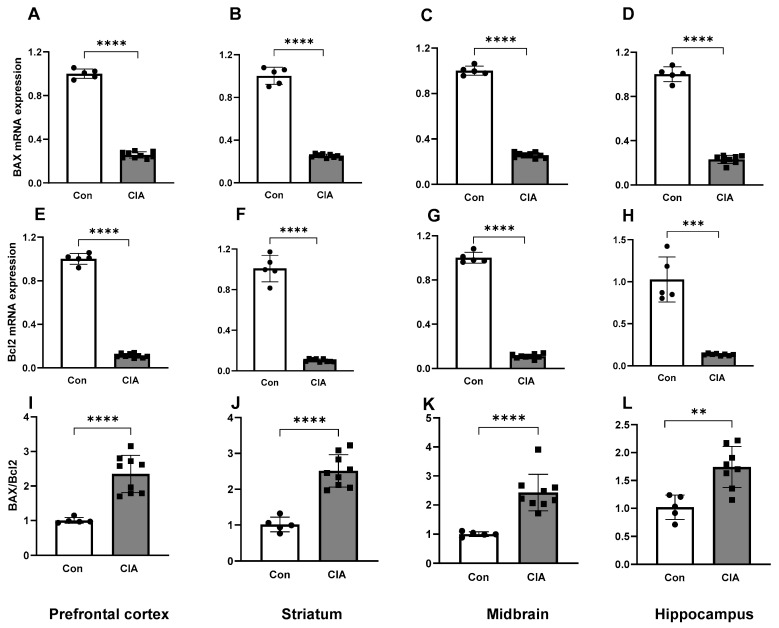
Apoptotic marker mRNA expressions in different brain regions in control (n = 5) and CIA (n = 9) rats. *BAX* mRNA expression in the prefrontal cortex (**A**), striatum (**B**), midbrain (**C**) and hippocampus (**D**). *Bcl2* mRNA expression in the prefrontal cortex (**E**), striatum (**F**), midbrain (**G**) and hippocampus (**H**). The ratio of *BAX/Bcl2* expression in the prefrontal cortex (**I**), striatum (**J**), midbrain (**K**) and hippocampus (**L**). Results are presented in arbitrary units normalized for the expression of the endogenous reference gene, *Tbp*. Data are represented as mean ± SD. ** *p* < 0.01, *** *p* < 0.001, **** *p* < 0.0001.

The mRNA expression of the pro-apoptotic marker *BAX* was increased in CIA rats compared to controls in all brain regions (all *p* < 0.01, Figure 2A–D). The mRNA expression of the anti-apoptotic marker *Bcl2* was decreased in the CIA rats compared to the control rats in all brain regions (all *p* < 0.001, Figure 2E–H). Nevertheless, the *BAX/Bcl2* ratio, an indicator of a pro-apoptotic state, was increased in all brain regions of the CIA group compared to controls (all *p* < 0.01, Figure 2I–L).

### 3.3. Neuroinflammation-Induced Decreases in Neurotropic Factor Expression

**Figure 3:**
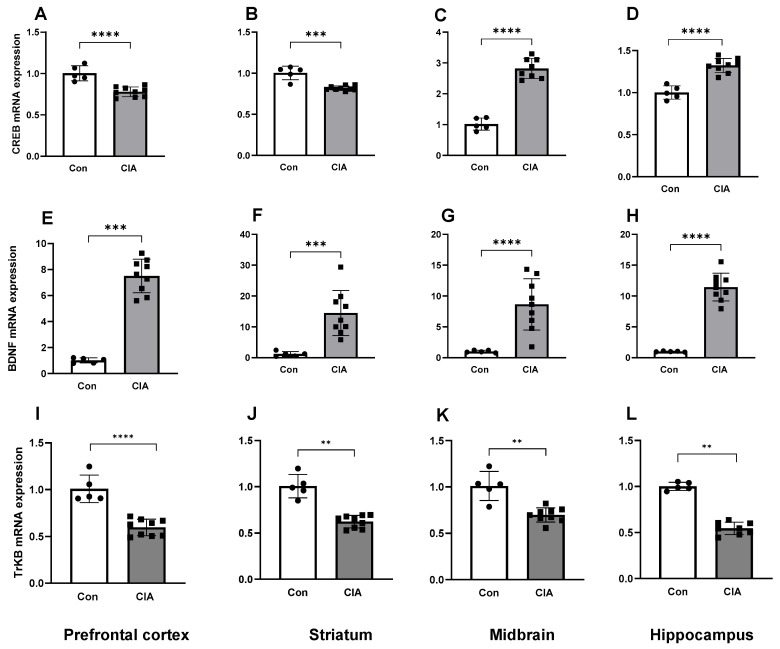
Neurotropic factor mRNA expression in different brain regions in control (n = 5) and CIA (n = 9) rats. *CREB* mRNA expression in the prefrontal cortex (**A**), striatum (**B**), midbrain (**C**) and hippocampus (**D**). *BDNF* mRNA expression in the prefrontal cortex (**E**), striatum (**F**), midbrain (**G**) and hippocampus (**H**). *TrkB* mRNA expression in the prefrontal cortex (**I**), striatum (**J**), midbrain (**K**) and hippocampus (**L**). Results are presented in arbitrary units normalized for the expression of the endogenous reference gene *Tbp*. Data are represented as mean ± SD. ** *p* < 0.01, *** *p* < 0.001, **** *p* < 0.0001.

In the CIA group, *CREB* mRNA expression was reduced compared to controls in the prefrontal cortex and striatum (both *p* < 0.01, Figure 3A,B); however, in the midbrain and hippocampus, *CREB* mRNA expression in CIA rats was increased compared to control rats (Figure 3C,D). Interestingly, *BDNF* mRNA expression was increased in CIA rats compared to controls in all brain regions (all *p* < 0.01, Figure 3E–H). Despite the *BDNF* upregulation, *TrkB* mRNA expression, the gene coding for the receptor for BDNF binding, was decreased in CIA rats compared to controls in all brain regions (all *p* < 0.05, Figure 3I–L).

### 3.4. Neuroinflammation-Induced Monoamine Dysregulation

**Figure 4:**
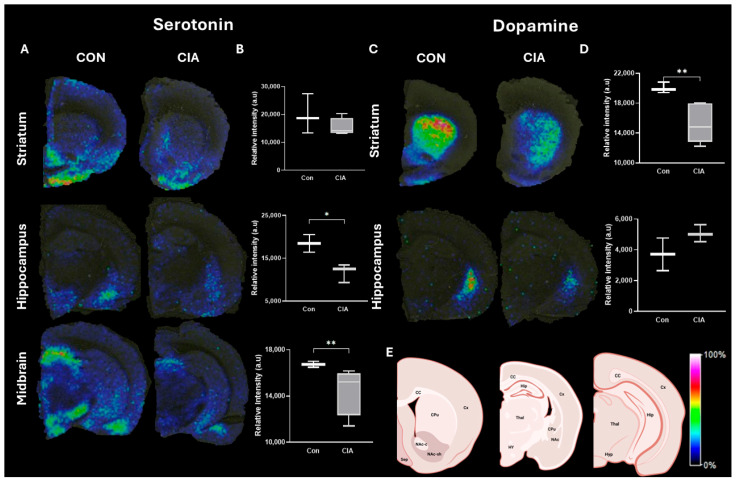
Neurotransmitter distribution and abundance in different brain regions in control and CIA rats (n = 3 per group). Representative images of serotonin (444.207 *m*/*z* ^†^) distribution (A) and the relative abundance (B) in the different brain regions of the control and CIA groups. Representative images of dopamine (674.286 *m*/*z* ^††^) distribution (C) and the relative abundance (D) in the different brain regions of the control and CIA groups. Histological reference (E). Key: Cx, cortex; CC, corpus callosum; LV, lateral ventricle; CPu, caudate putamen; NAc, nucleus accumbens; NAc-c, nucleus accumbens core; NAc-sh, nucleus accumbens shell; Hip, hippocampus; Thal, thalamus, Hy, hypothalamus; Sep, septum. Relative abundance is expressed as an arbitrary unit normalized using the root mean square and is represented as median (IQR). * *p* < 0.05, ** *p* < 0.01. ^†^ single derivatization with FMP-10; ^††^ double derivatization with FMP-10. Histological references created with BioRender.com.

In the CIA rats, the tissue distribution (Figure 4A) and relative abundance (Figure 4B) of serotonin were decreased compared to the control rats in both the hippocampus and the midbrain, but not in the striatum. In the CIA rats, the tissue distribution (Figure 4C) and relative abundance (Figure 4D) of dopamine were decreased compared to control rats in the striatum, but not in the hippocampus. These results suggest that CIA induced differential regional monoamine dysregulation.

## 4. Discussion

In the present study, we used the CIA rodent model to investigate the regional expression of neuroinflammatory markers, apoptotic and neurotrophic factors, as well as monoaminergic neurotransmission in the brain. We showed that collagen inoculation resulted in an increased mRNA expression of *IL-1β* in the brain, particularly in the prefrontal cortex, striatum and hippocampus, confirming the presence of neuroinflammation. This was coupled with an increase in the *BAX/Bcl2* ratio, suggesting a pro-apoptotic state in the CIA rats. Interestingly, the mRNA expression of the transcription factor, *CREB*, was decreased in the prefrontal cortex and striatum, while it was increased in the midbrain and the hippocampus. Despite the differential regional changes in *CREB* expression, mRNA expression of *BDNF*, a neurotrophic factor whose decrease is typically associated with neurodegeneration, was paradoxically increased in all brain regions, while the expression of its receptor *TrkB* was decreased in all brain regions. These results suggest differential, region-specific dysregulation of neurotrophic processes, which may be linked to neuroinflammation. Finally, we showed aberrant monoamine signalling, as there were decreases in serotonin in the hippocampus and the midbrain, but not the prefrontal cortex, while dopamine was decreased only in the striatum of CIA rats compared to controls. Taken together, our results show that collagen inoculation resulted in the dysregulation of several region-specific molecular pathways associated with neurological disorders.

In this study, we inoculated rats with collagen, emulsified in incomplete Freund’s adjuvant, to induce localized paw and systemic inflammation. Indeed, our laboratory has previously shown an increased arthritis paw score, as measured by the arthritis index and increases in circulating pro-inflammatory cytokines in this model [5,7]. Similar to previous reports in the CIA model [14], in the present study we showed increased mRNA expression of *IL-1β* in the brain of the CIA rats compared to controls, indicating neuroinflammation. Systemic inflammation, in the CIA model, has previously been shown to induce neuroinflammation via disfunction of the blood–brain barrier (BBB) [14,29]. The specialized microvascular endothelium of the BBB interacts closely with astrocytes, the immune cells of the brain, to maintain the integrity of the barrier. These authors suggested that when compromised, the barrier becomes more permeable to circulating factors such as inflammatory cytokines, resulting in an immune reaction in the brain [14]. Importantly, the increased *IL-1β* expression in the present study was shown in the prefrontal cortex, striatum and hippocampus, but not in the midbrain. This finding is significant in the study of neurological disorders, since their pathophysiology is often associated with deleterious changes in specific brain regions. Indeed, changes in the hippocampus, due to its high density of glial and other immune cells, is strongly implicated in mood disorders characterized by depressive-like symptoms [38]. Several studies have shown that increases in interleukin (IL)-1β expression are associated with neurodegeneration, multiple sclerosis, traumatic brain injury and diabetic neuropathy, which may explain the typical hippocampal volume loss seen in depressive patients [39]. Interestingly, in the present study we showed a significant 4-fold increase in the hippocampal mRNA expression of *IL-1β* when compared to the 2-fold increase in that in the prefrontal cortex and the striatum. The increased expression of *IL-1β* is congruent with the changes observed in the mRNA expression of apoptotic markers *BAX* and *Bcl2* in the present study. It is well established that activation of the IL pathways in the brain drives apoptotic cell death and neurodegeneration [39]. BAX is a potent pro-apoptotic protein localizing in the mitochondria, activating caspase 3, membrane blebbing, nuclear fragmentation and subsequent cell death [40]. Bcl2 is a crucial regulator of apoptosis, serving a protective function by blocking the release of cytochrome C which is required for the activation of caspases involved in apoptosis [40]. Interestingly, both *BAX* and *Bcl2* mRNA expression were decreased in all brain regions compared to controls; however, that ratio of *BAX/Bcl2* was increased in this study. It is well documented that an increase in the BAX/Bcl2 ratio serves as a cell death switch and reduces cellular resistance to apoptotic stimuli, leading to cell death [41]. These results suggest that the hippocampus is most susceptible to systemic inflammation and may lead to neurodegeneration that could be attributed to the neuroinflammation-induced changes in the *BAX/Bcl2* ratio.

Furthermore, the increased pro-apoptotic signals in the present study are substantiated by changes in neurotrophic factors observed in this study. In this regard, we measured the regional mRNA expression of the transcriptional regulator *CREB*, the neurotrophic factor *BDNF* and its receptor *TrkB*, as markers of neuronal regeneration, synaptic plasticity, neurogenesis and neuronal health [31,33]. Interestingly, *CREB* mRNA expression was decreased in the prefrontal cortex and striatum, while it was increased in the hippocampus and the midbrain. In contrast, in all brain regions *BDNF* mRNA expression was increased, while that of its receptor, *Trkb*, was decreased. CREB is a regulator of synaptic plasticity and neuronal health, via binding to the promoter region of the *BDNF* gene, and is an important target of several antidepressant therapies [42]. Decreases in BDNF expression are frequently associated with neurodegenerative disorders [33]. Indeed, a previous preclinical study, using a similar AIA rat model, showed decreased BDNF expression in the hippocampus and amygdala, which was reversed with anti-rheumatic drug therapy [35]. These differences in BDNF expression may be explained by several factors. While it has been suggested that BDNF expression is decreased in neurodegenerative disorders, some studies have proposed that it may be upregulated as a compensatory mechanism to protect against neuronal damage [43,44]. Hence, in the present study, the increased *BDNF* expression may be a result of the pro-apoptotic signals as shown in the increased *BAX/Bcl2* ratio. Furthermore, the different models and methodological design may result in differences in the expression of these factors. In this regard, BDNF expression may depend on the model induction and the duration of the study. In addition, in the present study we showed that *TrkB* mRNA expression was decreased, and hence to ensure adequate ligand–receptor interactions, *BDNF* expression may have been upregulated to account for the decreased receptor expression. Furthermore, the high density of immune cells in the hippocampus makes it the focal point of inflammatory changes in the brain and hence explains it susceptibility to neurological disorders with underlying neurodegenration [38]. Moreover, it has been suggested that there is a bi-directional relationship between CREB and BNDF [45]. Taken together, our results suggest that the increased *CREB* expression in the hippocampus may be a compensatory mechanism to preserve neuronal function in response to the increased *BDNF* expression and increased neuroinflammation and apoptotic markers. Interestingly, the lack of difference in the *IL-6* mRNA expression between control and CIA animals in the present study also supports the compensatory protective mechanism. Indeed, it has been suggested that IL-6 has a dual anti- and pro-inflammatory role in the brain [46]. In addition, IL-6 also plays an important neurogenic role, behaving in a neurotrophic manner [46]. The differential regional inflammatory cytokine expression in the brain and its relation to clinical presentation and symptomology require further investigation.

The changes in inflammatory mediators, apoptotic markers and neurotrophic factors in the present study suggest region-specific neurodegeneration in CIA-treated animals. Therefore, it is not surprising that we also observed decreases in serotoninergic and dopaminergic neurotransmission in the striatal, hippocampal and midbrain bregma levels, respectively. Similarly, others have shown that activation of the IL-1β pathway is strongly implicated in decreased dopamine neurotransmission [47]. Changes in brain monoamine signalling, particularly dopamine and serotonin, have been associated with depressive-like symptoms in various neurological disorders [48]. Taken together, our results highlight the central role of neuroinflammation in driving several pathophysiological processes and molecular pathways associated with neurological disorders.

Our findings suggest that there is a lack of concurrent increase in *TrkB* and *BDNF* expression. Therefore, further investigations using protein analysis to determine if the changes in mRNA expression in this study are indicative of functional changes in BDNF activity are required. Nevertheless, previous reports have shown an association between BDNF protein and mRNA expression [49,50]. In addition, future studies may benefit from spatial transcriptomic and RNAseq analysis to validate RT-qPCR findings and identify novel biomarkers of inflammation-induced neurological changes. We did not report neurobehavioral changes, hence the association between phenotypic changes and biomolecular markers should be determined in future studies. Lastly, several other intracellular factors have been shown to influence the regulation of BDNF and CREB expression [45]. Therefore, other factors not measured in the present study may influence the differential expression of these biomarkers in the brain, which should be investigated in future studies.

## 5. Conclusions

In the present study, CIA resulted in neuroinflammation which may have contributed to increases in a pro-apoptotic state in all brain regions. CIA inoculation also resulted in aberrant expression of neurotrophic factors and monoamine signalling. These results confirm that systemic inflammation may drive several region-specific brain molecular changes associated with neurodegenerative disorders.

## Data Availability

All data collected in this study are presented in this manuscript. Raw data may be made available upon reasonable request from the corresponding author (aletta.millen@wits.ac.za).

## Abbreviations

AIA: adjuvant-induced arthritis; BAX: bcl-2 like protein 4; Bcl-2: B-cell lymphoma 2; BDNF: brain-derived neurotrophic factor; CIA: collagen-induced arthritis; CC: corpus callosum; cDNA: complementary deoxyribonucleic acid; CPu: caudate putamen; CREB: cAMP binding response element; Cx: cortex; FMP-10: fluoromethylpyridinium; FT-ICR: Fourier-transform ion cyclotron resonance; IDO: indoleamine 2,3 dioxygenase; IL: interleukin; IL-1β: interleukin 1-beta; IL-6: interleukin-6; IQR: interquartile range; i.p.: intraperitoneal; Hip: hippocampus; HPA: hypothalamic–pituitary–adrenal; Hy: hypothalamus; LV: lateral ventricle; MALDI: matrix-assisted laser desorption ionisation; mRNA: messenger ribonucleic acid; MSI: mass spectrometry imaging; NAc: nucleus accumbens; NAc-c: nucleus accumbens core; NAc-sh: nucleus accumbens shell; RA: rheumatoid arthritis; RNA: ribonucleic acid; RMS: root mean square; RT-PCR: real-time polymerase chain reaction; SD: standard deviation; Sep: septum; Thal: thalamus; TrkB: tyrosine receptor kinase B.
